# Guidelines on Statistical Reporting at Nicotine & Tobacco Research

**DOI:** 10.1093/ntr/ntv131

**Published:** 2015-06-15

**Authors:** Marcus R. Munafò, E. Paul Wileyto

**Affiliations:** ^1^School of Experimental Psychology, University of Bristol, Bristol, United Kingdom;; ^2^Department of Biostatistics and Epidemiology, University of Pennsylvania, Philadelphia, PA

Despite the broad array of tools available, we in the biomedical sciences have become limited in the way we do statistical inference, with null hypothesis significance testing (NHST) the dominant approach and small *P* values the measure of our worth. However, there are a number of important limitations to NHST, at least as it is often implemented in practice, and these limitations are beginning to provoke a response across the literature in our field. One journal has even banned the use of NHST entirely.^1^ Here we describe some of these limitations, and offer recommendations for better reporting of statistical analyses that we would encourage our authors to adopt.

Tressoldi and colleagues^2^ describe three main limitations to NHST. First, NHST focuses on rejection of the null hypothesis at a prespecified level of probability (typically 5%, or .05). The implicit assumption, therefore, is that we are only interested answering “Yes!” to questions of the form “Is there a difference from zero?”. What if we are interested in cases where the answer is “No!”? Since the null hypothesis is hypothetical and unobserved, NHST doesn’t allow us to conclude that the null hypothesis is true. Second, *P* values can vary widely when the same experiment is repeated (eg, because the participants you sample will be different each time)—in other words, it gives very unreliable information about whether a finding is likely to be reproducible. This is important in the context of recent concerns about the poor reproducibility of many scientific findings.^3^ Third, with a large enough sample size we will always be able to find something to write about, although it may be a clinically meaningless or theoretically uninteresting relationship. No observed distribution is ever exactly consistent with the null hypothesis, and as sample size increases the likelihood of being able to reject the null increases. This means that trivial differences (eg, a difference in age of a few days) can lead to a *P* value less than .05 in a large enough sample, despite the difference having no practical or theoretical importance. The last point is particularly important, and relates to two other limitations. Namely, the *P* value doesn’t tell us anything about how large an effect is (ie, the effect size), or about how precise our estimate of the effect size is. Any measurement will include a degree of error, and it’s important to know how large this is likely to be. Fortunately, these limitations can be addressed, at least in part, by simple changes to the way we report and interpret the results of our statistical analyses.

One is the routine reporting of effect size and confidence intervals. The confidence interval is essentially a measure of the reliability of our estimate of the effect size, and can be calculated for different ranges. A 95% confidence interval, for example, represents the range of values that contains the true effect size in the underlying population 95% of the time. Reporting the effect size and associated confidence interval therefore tells us both the magnitude of the observed effect, and the degree of precision associated with that estimate. The reporting of effect sizes and confidence intervals is recommended by a number of scientific organizations, including the American Psychological Association, and the International Committee of Medical Journal Editors.

Another is to adopt a more Bayesian perspective in the interpretation of analyses (without necessarily fully adopting Bayesian methods of statistical inference). The conventional Frequentist classification of results from statistical tests into “significant” and “nonsignificant” is based strictly on the null hypothesis, and is made without reference to the distribution of the alternative hypothesis. Under the null hypothesis, the *P* value is distributed as a continuous uniform random variable. Bayesians can become nearly apoplectic trying to explain that the *P* value only has meaning when interpreted using the prior distribution of the alternative hypothesis. Ioannidis^4^ has suggested using the positive predictive value as a measure of the importance of a positive finding. This is measure of the fraction of positive results that are truly positive, derived from epidemiological concepts of sensitivity and specificity, where we substitute 1−β (ie, power) for sensitivity and 1−α for specificity. Both a low prior probability for the alternative hypothesis (eg, exploratory research or unplanned sub-group analyses) and low statistical power (eg, small studies) reduce the positive predictive value, and therefore the likelihood that a statistically significant (ie, *P* < .05) result is actually true.^5^

We encourage our authors to take a more considered approach to statistical inference, and in particular to consider the use of measures of effect size and precision (eg, confidence) when reporting results of statistical tests, and to avoid reference to statistical “significance”. Another advantage of this approach is that it obviates the need for some of the tortured language we see used to describe *P* values in the .05 to .10 range (eg, https://mchankins.wordpress.com/2013/04/21/still-not-significant-2/). Instead, results can be reported with reference to effect size and precision, and interpreted in the context of not only the strength of evidence against the null hypothesis indicated by the *P* value obtained, but also the prior probability for the alternative hypothesis and the likely statistical power of the study. In our view, this approach will provide our readers with a more complete and nuanced interpretation of the results reported in our journal.

## Declaration of Interests

*None declared.*
